# On the Analogy and Differences between Tubercle and Scrofula

**Published:** 1849-09

**Authors:** 


*On the Analogy and Differences between Tubercle and Scrofula.*—M. A. Legrand concludes an elaborate and valuable series of papers, extending through several Nos. of the *Revue Médicale*, for last year, the fruit of many years' research, he informs us, with the following summary of the results at which he has arrived:

1. There undoubtedly exist analogies, which we may call symptomatic, between tubercle and scrofula; that is to say, one of these two morbid principles, the tubercular, may exhibit itself by symptoms which appear to belong to the other; but this is not the case with regard to the latter.

2. Tubercle possesses, so to speak, its morbid individuality, its molecular element—the tubercular globule—which is often met with in the scrofulous manifestations of tubercle.

3. Scrofula is always deficient in the morbid molecular element, and its existence is only proved by the constancy of the effects which are attributed to it.

4. The chief, or even the only seat of tubercle is in the internal organs, and the external manifestations of the morbid principle irradiate from the centre to the circumference.

5. Scrofula comports itself quite otherwise, and manifesting itself on the skin or periosteum, irradiates thence towards the internal organs, which, however, it never disorganizes in the same manner as tubercle.

6. Tubercle, in spite of the impoverishment of the blood it always induces, does not destroy, at least in the early periods, the inflammatory element, the fibrine, which well explains the occurrence of the phlegmasiæ, which so often complicate it, and which always hasten its disorganizing progress.

7. Scrofula likewise impoverishes the blood, but at the same time it seems to annihilate the inflammatory element. Thus inflammations rarely complicate it, and when such complication does exist, it often favors the cure of the disease.

8. All the changes observed in the blood and urine of tuberculous and scrofulous patients, are evidently consecutive; and they cannot be considered as the cause of these two diseases, whose principle is nevertheless very probably contained in the blood.

9. Finally, tubercle is never curable, or at least such cure constitutes a rare exception, while scrofula is almost always curable.—*Revue Médicale*, Nov., 1848.

